# The splicing modulator sudemycin induces a specific antitumor response and cooperates with ibrutinib in chronic lymphocytic leukemia

**DOI:** 10.18632/oncotarget.4212

**Published:** 2015-06-08

**Authors:** Sílvia Xargay-Torrent, Mónica López-Guerra, Laia Rosich, Arnau Montraveta, Jocabed Roldán, Vanina Rodríguez, Neus Villamor, Marta Aymerich, Chandraiah Lagisetti, Thomas R. Webb, Carlos López-Otín, Elias Campo, Dolors Colomer

**Affiliations:** ^1^ Experimental Therapeutics in Lymphoid Malignancies Group, Institut d'Investigacions Biomèdiques August Pi i Sunyer (IDIBAPS), Barcelona, Spain; ^2^ Hematopathology Unit, Department of Pathology, Hospital Clinic, University of Barcelona, Institut d'Investigacions Biomèdiques August Pi i Sunyer (IDIBAPS), Barcelona, Spain; ^3^ Center for Chemical Biology, Biosciences Division, SRI International, Menlo Park, California, USA; ^4^ Departamento de Bioquímica y Biología Molecular, Universidad de Oviedo - IUOPA, Oviedo, Spain

**Keywords:** SF3B1, chronic lymphocytic leukemia, sudemycin, ibrutinib, spliceosome

## Abstract

Mutations or deregulated expression of the components of the spliceosome can influence the splicing pattern of several genes and contribute to the development of tumors. In this context, we report that the spliceosome modulator sudemycin induces selective cytotoxicity in primary chronic lymphocytic leukemia (CLL) cells when compared with healthy lymphocytes and tumor cells from other B-lymphoid malignancies, with a slight bias for CLL cases with mutations in spliceosome-RNA processing machinery. Consistently, sudemycin exhibits considerable antitumor activity in NOD/SCID/*IL2Rγ*−/− (NSG) mice engrafted with primary cells from CLL patients. The antileukemic effect of sudemycin involves the splicing modulation of several target genes important for tumor survival, both in *SF3B1*-mutated and -unmutated cases. Thus, the apoptosis induced by this compound is related to the alternative splicing switch of *MCL1* toward its proapoptotic isoform. Sudemycin also functionally disturbs NF-κB pathway in parallel with the induction of a spliced *RELA* variant that loses its DNA binding domain. Importantly, we show an enhanced antitumor effect of sudemycin in combination with ibrutinib that might be related to the modulation of the alternative splicing of the inhibitor of Btk (*IBTK*). In conclusion, we provide first evidence that the spliceosome is a relevant therapeutic target in CLL, supporting the use of splicing modulators alone or in combination with ibrutinib as a promising approach for the treatment of CLL patients.

## INTRODUCTION

Chronic lymphocytic leukemia (CLL) is the most frequent adult leukemia in western countries. The tumor is characterized by the proliferation and progressive accumulation of distinctive mature clonal B lymphocytes in blood, bone marrow and lymphoid tissues. The prognosis of the patients is highly heterogeneous; while some show an indolent disease, others follow an aggressive course with a median survival of less than 4–5 years [1, 2]. These clinical differences are largely associated with the mutational status of the variable region of immunoglobulin heavy chain genes (*IGVH*), cytogenetic alterations and expression of ZAP-70, CD38 or CD49d [3, 4].

In the last years, whole-genome and exome sequencing have revealed the presence of novel recurrent somatic mutations with functional impact in CLL patients. These studies have uncovered genetic alterations that tend to cluster in different pathways, including Notch signaling, RNA splicing and processing machinery, innate inflammatory response, Wnt signaling and telomere maintenance [5–8]. *SF3B1* is among the most frequently mutated genes in CLL affecting up to 5–18% of patients [6, 8, 9]. This gene encodes a core component of the spliceosome, the machinery that catalyzes the removal of introns from precursor mRNAs and ensures the fidelity of the process. Consistent with the crucial role of this pathway in CLL, mutations in other genes of the mRNA splicing, processing and transport machinery are also found to be frequently mutated in CLL patients [10].

Clinically, *SF3B1* mutations provide independent prognostic information from the other known CLL prognostic markers. Such mutations correlate with a more aggressive behavior of the disease, rapid tumor progression, poorer clinical outcome and shorter time to treatment as well as treatment refractoriness [6, 8, 9, 11–13]. Consistently, *SF3B1* mutations are mostly subclonal events in CLL, and therefore likely involved in disease progression [14].

Besides mutations, overexpression of the components of the spliceosome has been found in cancer, as a result, targeting this machinery has been proposed as a relevant therapeutic target [15]. In this context, several bacterial natural products have been described that target SF3B1 and modulate RNA splicing, including FR901464 and pladienolide B, which induce antitumor activity in different tumor types [16, 17]. Additionally, the pladienolide derivative E7107 entered clinical trials for solid tumors. However its development has been significantly hampered due to the structural complexity of the molecule [18, 19]. A novel set of totally synthetic analogs of FR901464, the sudemycins, have demonstrated cytotoxicity toward several human tumor models, including lymphoma, both *in vitro* and in xenograft models [20–22].

Therefore, the spliceosome is an important emerging target for CLL therapy that has recently been uncovered but remains to be significantly exploited. Herein, we evaluate for the first time the antitumor effect of the spliceosome modulator sudemycin in *SF3B1*-mutated and -unmutated CLL cells in the *in vitro* and *in vivo* settings. Moreover, we investigate the molecular mechanisms underlying the antitumor effect of sudemycin in these cells and explore the effects of its combination with the clinically relevant BTK inhibitor, ibrutinib.

## RESULTS

### Sudemycins induce selective cytotoxicity in CLL cells with enhanced response in cases with mutations in the splicing and RNA processing machinery

**Figure 1:**
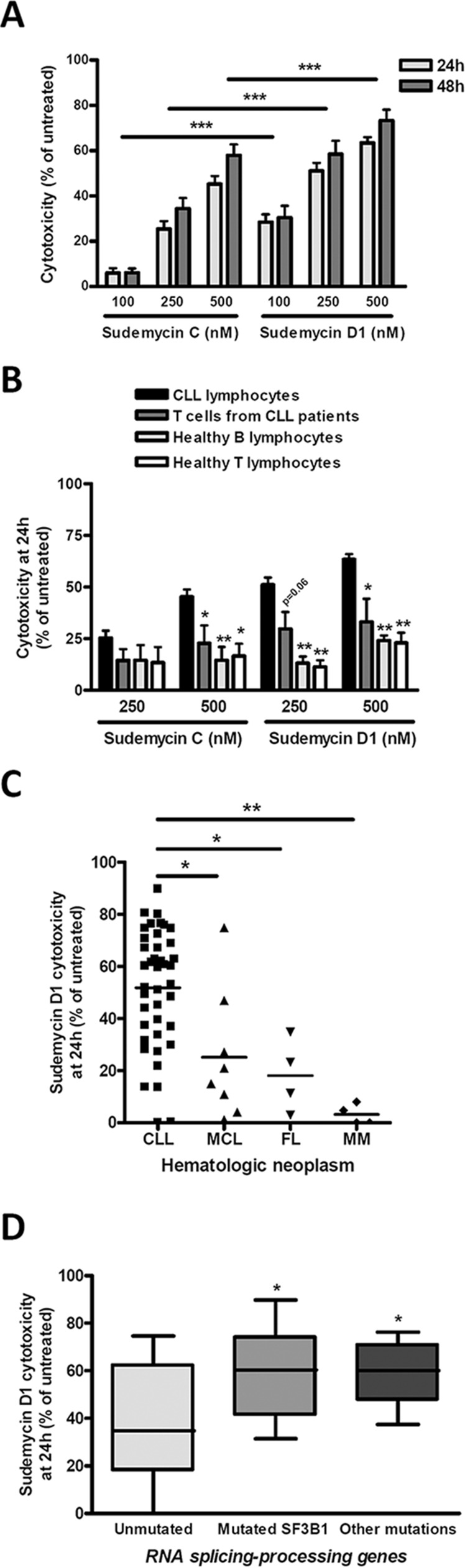
Sudemycins selective induction of cytotoxicity in CLL **A.** Primary cells from CLL patients were incubated with the indicated doses of sudemycin C and D1 for 24 (*n* = 41) and 48 hours (*n* = 27). Cytotoxicity was quantified after annexin V/PI staining by flow cytometry and referred to the untreated control. Bars represent the mean ± SEM of all samples analyzed. ***p < 0.001. **B.** CLL cytotoxicity after sudemycin (500 nM) exposure for 24 hours was compared to that of B and T lymphocytes from healthy donors (*n* = 5) as well as T lymphocytes from CLL patients (*n* = 4). T and B lymphocytes were labeled and gated with anti-CD3-FITC and anti-CD19-PE antibodies. Mean ± SEM of all cases analyzed. *p < 0.05, **p < 0.01. **C.** Sudemycin D1-induced cytotoxicity (250 nM) was compared in CLL (*n* = 41), MCL (*n* = 8), FL (*n* = 4) and MM (*n* = 4) samples. Cell death was measured at 24 hours by flow cytometry and referred to the corresponding untreated cells. Mean ± SEM of all cases analyzed. *p < 0.05, **p < 0.01. **D.** Diagram representing cytotoxicity at 24 hours of sudemycin D1 (250 nM) in CLL samples classified according to the presence of *SF3B1* or other mutations in splicing-RNA processing machinery genes (Unmutated, *n* = 16; *SF3B1*-mutated, *n* = 14; other mutations *n* = 8). Cytotoxicity was referred to the respective untreated control. *p < 0.05.

To evaluate sensitivity to sudemycins, tumor cells from 41 CLL patients (Table 1) were tested with sudemycin C and the new improved version sudemycin D1 [22]. Cytotoxicity was measured after incubation of CLL cells with 100, 250 and 500 nM of drugs for 24 hours (Figure 1A). Sudemycin C and D1 induced apoptosis in CLL cells in a dose-response fashion achieving a mean response of 45.2 ± 20.1% and 63.4 ± 15.3% at the highest dose, respectively. At 48 hours, the cytotoxic effect was enhanced and sudemycin-induced apoptosis reached 57.9 ± 24.8% and 73.3 ± 24.3% for C and D1 at 500 nM dose. As expected, sudemycin D1 demonstrated to be more effective than C in inducing CLL apoptosis at all the doses tested and both timepoints (***p < 0.001, Figure 1A).

**Table 1:** Characteristics of CLL samples

Patient n°.	Age at diagnosis	Gender*	Binet/Rai stage	Previous treatment^#^	%CD19/CD5^†^	*IgVH* status^‡^	*SF3B1* status^‡^	*SF3B1* mutation	Other splicing-RNA processing machinery gene mutations	Cytogenetic alterations (FISH)^§^	% Cytotoxicity Sudemycin D1 250nM (24 h)
**CLL 1**	56	F	A/0	no	94	M	UM	no	no	13q del	27.3
**CLL 2**	67	M	A/0	no	94	M	UM	no	no	13q del	49.3
**CLL 3**	44	M	C/IV	no	97	M	UM	no	no	13q del	39.6
**CLL 4**	62	F	A/0	no	96	M	UM	no	no	11q del	0.0
**CLL 5**	59	M	B/II	no	95	M	UM	no	no	13q del	0.2
**CLL 6**	61	M	C/III	no	96	UM	UM	no	no	11q del	62.8
**CLL 7**	52	M	B/II	R-FCM; R-BDM	97	UM	UM	no	no	normal	68.9
**CLL 8**	71	M	C/IV	no	94	M	UM	no	no	13q del	74.6
**CLL 9**	66	F	A/0	no	84	M	UM	no	no	13q del	13.6
**CLL 10**	37	M	A/I	Fludarabine	64	M	UM	no	no	normal	62.9
**CLL 11**	58	M	B/II	FCM	95	UM	UM	no	no	ND	62.1
**CLL 12**	63	M	C/IV	R-FCM	97	UM	UM	no	no	13q del; 17p del	61.6
**CLL 13**	58	M	A/0	no	95	M	UM	no	no	normal	21.8
**CLL 14**	51	M	A/0	no	80	M	UM	no	no	13q del; 11q del	13.7
**CLL 15**	74	M	C/IV	FC	93	UM	UM	no	no	normal	28.2
**CLL 16**	74	M	B/II	R-FCM	87		UM	no	ND	normal	51.9
**CLL 17**	64	M	B/II	no	93	M	UM	no	no	trisomy 12	29.9
**CLL 18**	44	M	C/IV	no	98		UM	no	ND	13q del	80.0
**CLL 19**	78	M	A/0	no	95	M	UM	no	ND	trisomy 12	80.6
**CLL 20**	47	M	C/IV	2CdA; FCM; Campath; CHOP	93	ND	M	G742D	no	13q del	72.5
**CLL 21**	49	M	A/I	FCM; R-CHOP; R-FCM	92	UM	M	K700E	no	13q del	31.5
**CLL 22**	71	M	B/II	no	93	UM	M	R625H	no	normal	60.3
**CLL 23**	70	M	B/II	no	95	UM	M	G742D	no	13q del	60.3
**CLL 24**	63	M	A/0	no	90	M	M	M757T	no	normal	75.9
**CLL 25**	70	F	B/I	FC	99	M	M	K700E	no	11q del; 13q del	70.7
**CLL 26**	54	F	B/I	FCM; CHOP-like	89	UM	M	R625H	no	11q del	45.2
**CLL 27**	47	M	B/II	FCM; CHOP	79	UM	M	K741N	no	normal	60.7
**CLL 28**	48	M	B/II	CHOP	88	UM	M	G742D	no	ND	89.8
**CLL 29**	45	M	B/II	CHOP; FCM	95	ND	M	G742D	no	ND	59.(Continued )5
**CLL 30**	53	F	B/II	Fludarabine; R-FCM	70	UM	M	K666E	no	11q del; 13q del	76.5
**CLL 31**	56	M	B/II	Chlorambucil	99	UM	M	T663I	no	13q del	33.8
**CLL 32**	56	M	B/III	no	95	M	M	E862K	no	13qdel; trisomy 12	48.4
**CLL 33**	78	F	B/II	no	94	UM	M	H662D	no	13qdel; 11q del	36.9
**CLL 34**	86	F	A/0	no	87	UM	UM	no	SRSF7 (U2 no SF3B1)	trisomy 12	44.0
**CLL 35**	71	M	A/0	no	97	UM	UM	no	XPO1 (RNA transport)	normal	67.1
**CLL 36**	45	F	B/II	R-FCM	86	UM	UM	no	NXF1 (RNA transport)	trisomy 12; 17p del	37.4
**CLL 37**	78	M	B/II	no	95	UM	UM	no	DDX3X (RNA transport)	13q del	74.7
**CLL 38**	66	M	C/IV	no	95	M	UM	no	CPEB3 (mRNA decay)	13q del	53.2
**CLL 39**	73	F	A/0	no	97	M	UM	no	SMG7 (mRNA decay)	normal	67.1
**CLL 40**	70	F	A/I	no	90	M	UM	no	CDC5L (Prp19 complex)	ND	76.3
**CLL 41**	38	M	A/0	no	98	M	UM	no	HELZ (RNA helicase), PIWIL3 (RNA silencing)	13q del; 11q del	51.0

ND: not determined

* M: male; F: female.

† quantified by flow cytometry

‡ UM: unmutated; M: mutated

§ assessed by FISH. del: deletion

# R: Rituximab; FCM: Fludarabine, Cyclophosphamide, Mitoxantrone; BDM: Bendamustine; 2CdA: Cladribine; CHOP: Cyclophosphamide, Doxorubicin, Vincristine, Prednisone; FC: Fludarabine, Cyclophosphamide

In parallel, we investigated the selective cytotoxicity of these compounds for CLL tumor cells. For this purpose we exposed B and T lymphocytes from healthy donors as well as T cells from CLL patients to the highest dose (500 nM) of both drugs. As shown in Figure 1B, sudemycins were significantly less cytotoxic in normal T and B counterparts than in CLL cells (*p < 0.05, **p < 0.01). Similar results were obtained with 250 nM dose. Sudemycin D1 (250 nM) was selected for further experiments because it was more effective at inducing apoptosis than C and maintained high selectivity for CLL cells.

Next, we sought to determine whether other B-lymphoid malignancies were also responsive to sudemycin D1. Tumor cells from mantle cell lymphoma (MCL; *n* = 8), follicular lymphoma (FL; *n* = 4) and multiple myeloma (MM; *n* = 4) were exposed to sudemycin D1 for 24 hours (Supplementary Table S1) and cytotoxicity was compared to that of CLL cells. Apoptosis induction resulted to be significantly higher in CLL cells than in tumor cells from the other malignancies studied, indicating a particular sensitivity of CLL cells to sudemycin D1 (Figure 1C; *p < 0.05, **p < 0.01).

Finally, we carefully examined CLL samples according to the presence of *SF3B1* mutations. In addition, we also considered mutations in genes from other components of the splicing and RNA processing machinery. Of 38 CLL cases, 14 were *SF3B1*-mutated, 8 harbored mutations in other genes of the splicing and RNA processing machinery and 16 were unmutated for both conditions (Table 1). Interestingly, although all cases were particularly sensitive to sudemycin D1, the mutated *SF3B1* CLL cells were more sensitive to the drug than the unmutated ones. Intriguingly, those cases with other mutations in splicing and RNA processing machinery genes were as sensitive as *SF3B1*-mutated cases to the spliceosome modulator (Figure 1D, *p < 0.05).

### The number of CLL cells in the peripheral blood and spleen decreases in sudemycin D6-treated mice

**Figure 2:**
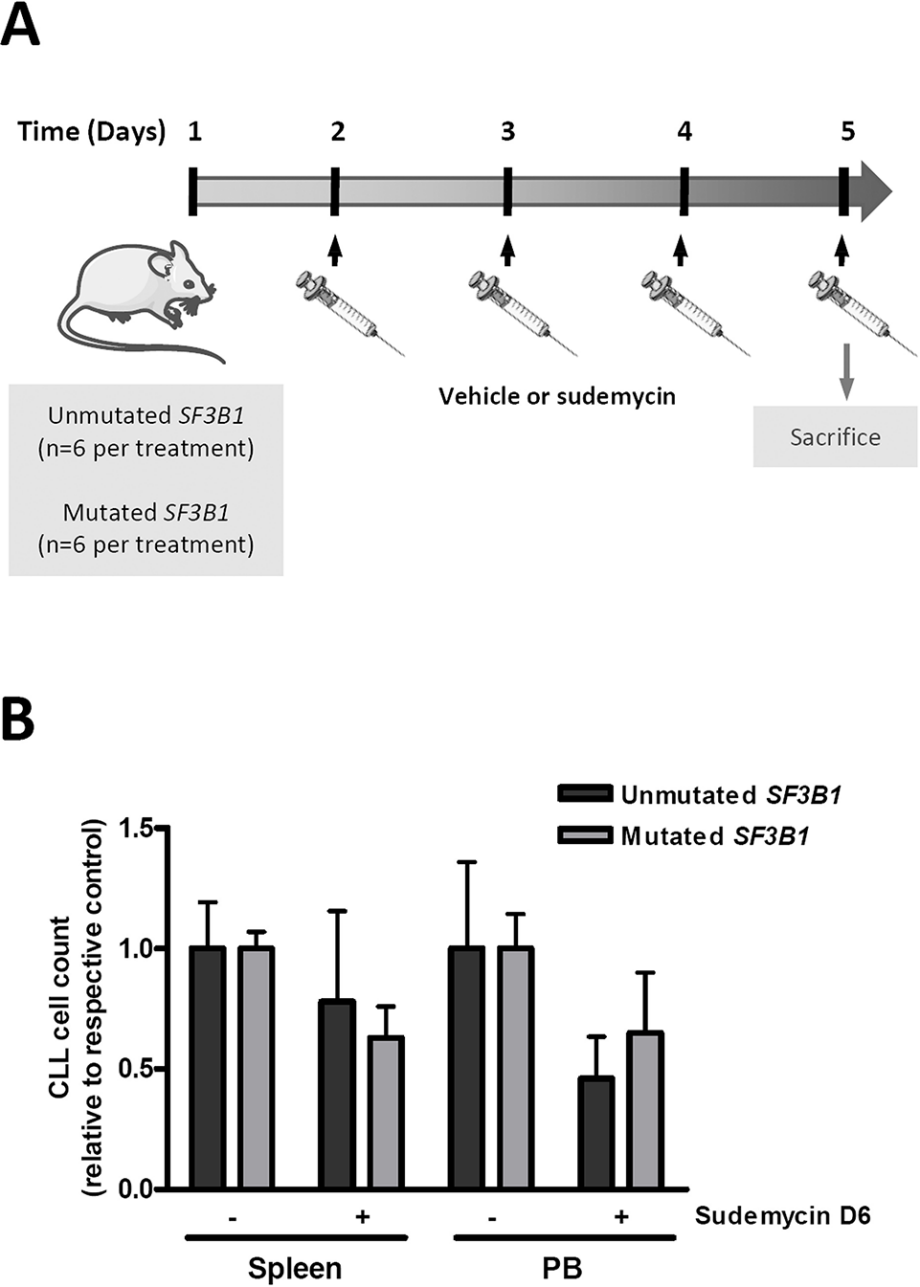
Effect of sudemycin D6 in an adoptive transfer model of CLL **A.** CLL cells were inoculated via the tail in NSG mice. After 24 hours, mice were treated with vehicle or sudemycin D6 (14 mg/kg) daily during 4 days and sacrificed. **B.** PB and spleen samples were recovered and viable CLL cells in these compartments were counted by flow cytometry. Bars represent the mean ± SEM of *SF3B1*-unmutated (*n* = 6 per group) and -mutated (*n* = 6 per group) cases.

In view of the significant *in vitro* antitumor activity of sudemycin, the drug was accessed for efficacy in NOD/SCID/*IL2Rγ*−/− (NSG) mice engrafted with primary cells from CLL patients. For these studies sudemycin D6 was used, which exhibited exactly the same cytotoxicity as D1 (data not shown) and had been optimized for *in vivo* activity and dosing [22]. CLL cells from *SF3B1*-unmutated and -mutated patients were intravenously inoculated via the tail in the mice (*n* = 6 per condition). Since the *in vivo* antitumor effect of sudemycin D6 had been shown to be very fast and already significant after 5 days of treatment [22], mice were treated with vehicle or sudemycin D6 (14 mg/kg) daily for 4 days and sacrificed (Figure 2A). As shown in Figure 2B, sudemycin D6 was able to reduce the presence of CLL cells in the peripheral blood (PB) and spleen of the mice. Tumor cells reduction in the spleen was near 25 and 40% in *SF3B1*-unmutated and -mutated cases, respectively. The decrease of CLL cells in PB samples was about 50 and 40% for *SF3B1*-unmutated and -mutated cases, correspondingly. Altogether these results confirmed that sudemycin D6 was effective in this NSG/CLL xenograft model.

### The apoptotic effect of sudemycin D1 is related to *MCL1* alternative splicing

**Figure 3:**
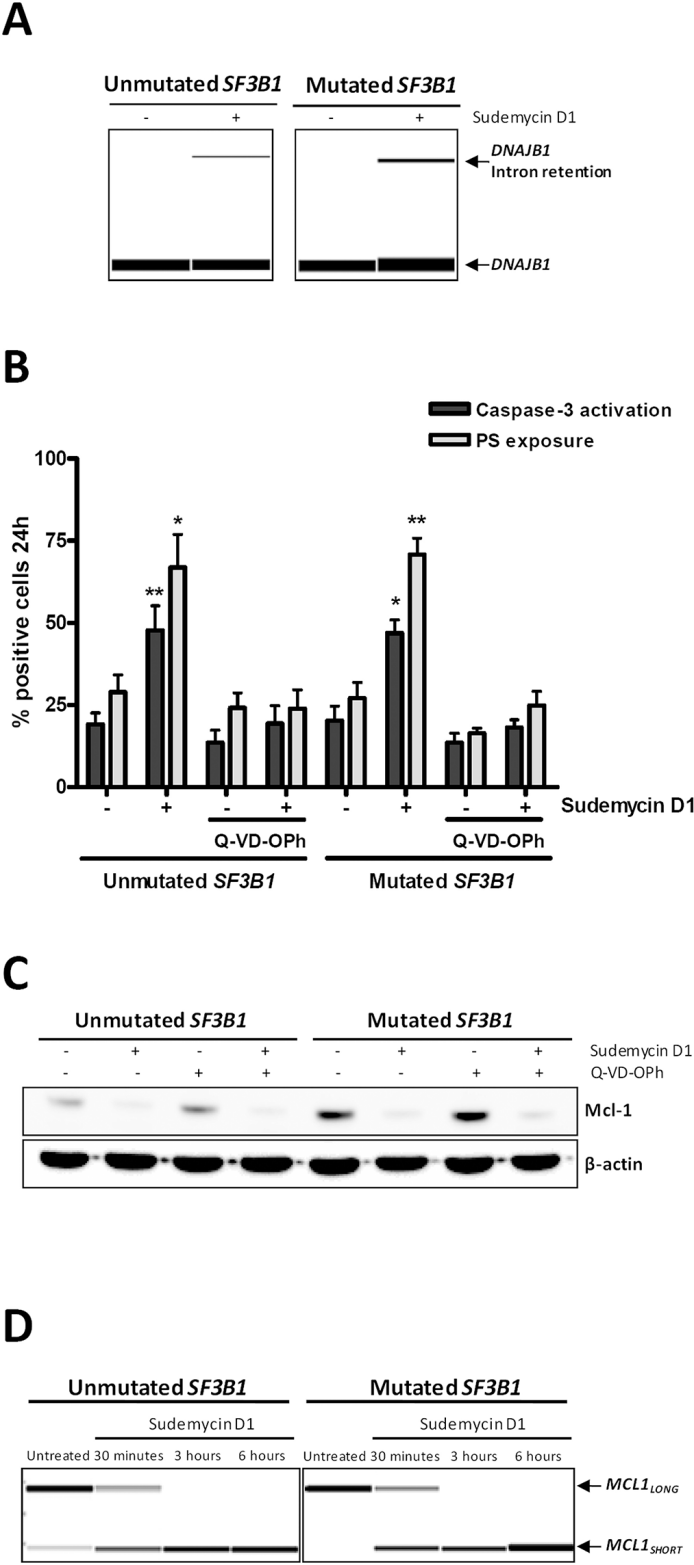
Sudemycin-induced *MCL1* alternative splicing **A.** RT-PCR analysis of spliced and unspliced *DNAJB1* mRNAs. cDNA was obtained from sudemycin D1-treated CLL cells (250 nM) for 6 hours and RT-PCR was performed with primers spanning two exons; products were analyzed in a QIAxcel capillary electrophoresis device. **B.** Analysis of apoptosis in CLL samples exposed to sudemycin D1 (250 nM; 24 hours) and/or the caspase inhibitor Q-VD-OPh (10 μM). Caspase 3 and PS exposure levels were quantified by flow cytometry by staining of cells with CellEvent caspase 3/7 green assay and annexin V-FITC, respectively. Bars represent the mean ± SEM of *SF3B1*-unmutated (*n* = 4) and -mutated (*n* = 5) cases. *p < 0.05; **p < 0.01. **C.** Mcl-1 protein levels evaluated by western blot after sudemycin D1 (250 nM; 24 hours) and/or Q-VD-OPh (10 μM) exposure in CLL primary cells. **D.** RT-PCR analysis of *MCL1* splicing in sudemycin D1-treated CLL cells (250 nM) for the indicated times. PCR was performed with primers binding exons 1 and 3, and products were analyzed in a QIAxcel capillary electrophoresis device.

To assess the ability of sudemycin D1 to modulate spliceosome activity in CLL cells, we analyzed *DNAJB1* expression as a surrogate marker for RNA splicing modulation [16]. A 6-hour treatment was chosen to avoid unspecific events related to cell death induction. As shown in Figure 3A, we confirmed that a 6-hour sudemycin D1 incubation increased the unspliced form of *DNAJB1* that retained intron 2, both in *SF3B1*-unmutated and -mutated CLL samples.

Next, in order to characterize the cell death mechanisms of sudemycin D1-induced cytotoxicity, primary cells from *SF3B1*-unmutated and -mutated CLL cases were preincubated with the pan-caspase inhibitor Q-VD-OPh before drug treatment and apoptotic markers were analyzed by flow cytometry. As shown in Figure 3B, Q-VD-OPh efficiently blocked both caspase-3 activation and phosphatidylserine (PS) exposure, indicating that the apoptotic process was caspase-dependent. Interestingly, sudemycin D1 induced a marked downregulation of the antiapoptotic protein Mcl-1, which was not reverted by caspase-inhibitor treatment and thus not degraded by caspases (Figure 3C). Since *MCL1* gene encodes the antiapoptotic Mcl-1-_LONG_ and the proapoptotic Mcl-1-_SHORT_ proteins, and the Mcl-1-_LONG_/Mcl-1-_SHORT_ balance is regulated by alternative splicing [23], we hypothesized that sudemycin D1-induced apoptosis might be related to the switch of the alternative splicing of *MCL1* toward its proapoptotic short isoform. Using RT-PCR with specific primers for exon 1 and exon 3 of *MCL1*, we showed that sudemycin D1 upregulated *MCL1*-_SHORT_ splice variant, which lacks exon 2, while decreasing *MCL1*-_LONG_, already at 30 minutes of treatment, both in *SF3B1*-unmutated and -mutated CLL cases (Figure 3D).

### Sudemycin D1 induces *RELA* alternative splicing together with the inhibition of the NF-κB pathway

Among the prosurvival pathways in CLL, we were particularly interested in investigating whether sudemycins were able to modulate NF-κB signaling due to its critical role in CLL pathogenesis [24, 25].

**Figure 4:**
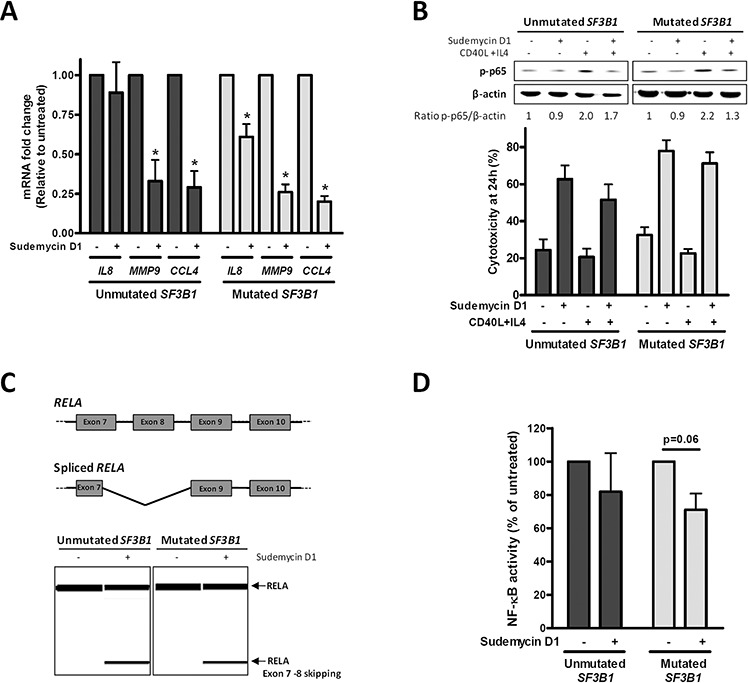
NF-κB pathway inhibition by sudemycin **A.** Gene expression analysis of NF-κB target genes (*IL8*, *MMP9* and *CCL4*) by quantitative real time PCR. Primary CLL cells from *SF3B1*-unmutated (*n* = 5) and mutated (*n* = 5) cases were incubated with sudemycin D1 (250 nM) for 6 hours. Gene expression levels are referred to the untreated control sample and represented as the mean ± SEM. *p < 0.05. **B.** *SF3B1*-unmutated (*n* = 5) and -mutated (*n* = 5) CLL samples were stimulated with CD40L (1 μg/mL) and IL4 (20 ng/mL) for 1 hour previously to sudemycin D1 incubation. Western blot analysis of p-p65 levels was performed after 6 hours of sudemycin D1 (250 nM) exposure (Upper panel). Cytotoxicity was analyzed by flow cytometry at 24 hours (Lower panel). **C.** Diagram representing alternative splicing on RELA exons 7–10 (Upper panel). Representative cases of RT-PCR analysis of spliced and unspliced *RELA* after CLL cells incubation with sudemycin D1 (250 nM) for 6 hours. Primers were designed against exon 7 and 10 from *RELA* and products were analyzed in a QIAxcel capillary electrophoresis device (Lower panel). **D.** NF-κB p65 activity was determined in *SF3B1*-unmutated (*n* = 3) and -mutated (*n* = 4) CLL cases exposed to sudemycin D1 (250 nM) for 6 hours as detailed in “Materials and Methods”.

First, gene expression of well-known protumor NF-κB target genes (*IL8*, *MMP9* and *CCL4*) was determined by quantitative RT-PCR. After a 6-hour exposure to sudemycin D1, a significant decrease in all NF-κB target genes was observed in both *SF3B1*-mutated and -unmutated CLL cases (*p < 0.05). *SF3B1*-mutated cases, showed a higher reduction of these targets than unmutated ones, suggesting that NF-κB modulation by sudemycin could, at least partially, explain the slight differences in sensitivity to sudemycin (Figure 4A).

To further explore the role of NF-κB on sudemycin activity, we stimulated the NF-κB pathway in CLL cells with CD40 ligand (CD40L) and interleukin 4 (IL4) soluble factors. Consistently, an about a 2-fold increase in phospho-p65 (p-p65) levels was detected after this stimulation. Sudemycin D1 demonstrated to overcome the prosurvival effects induced by CD40L and IL4 since it was as effective at inducing apoptosis as in control cells (Figure 4B).

Next, we focused on *RELA* (p65) because the functional effect of its alternative splice variants had been characterized. *RELA* exon 8 encodes for the Rel homology domain, which is required for dimerization and DNA binding [26]. In order to find whether this exon was lost by alternative splicing after sudemycin treatment, we analyzed by RT-PCR exons 7 to 10 from *RELA*. Indeed, an isoform lacking exon 8 and partially exon 7 (as confirmed by sequencing) was detected when CLL cells were exposed to a short incubation with sudemycin D1 in both *SF3B1*-mutated and unmutated cases (Figure 4C and Supplementary Information).

Finally, NF-κB activity was assessed in nuclear extracts from sudemycin-treated CLL cells both in *SF3B1*-mutated and unmutated cases by means of the p65 DNA binding capacity. As shown in Figure 4D, 6-hour exposure to sudemycin D1 reduced NF-κB p65 activity by 20–30%, showing a moderate bias for the *SF3B1*-mutated when compared to the -unmutated CLL cases.

### The combination of sudemycin D1 with ibrutinib results in an enhanced cytototoxic effect in parallel with *IBTK* splicing modulation

**Figure 5:**
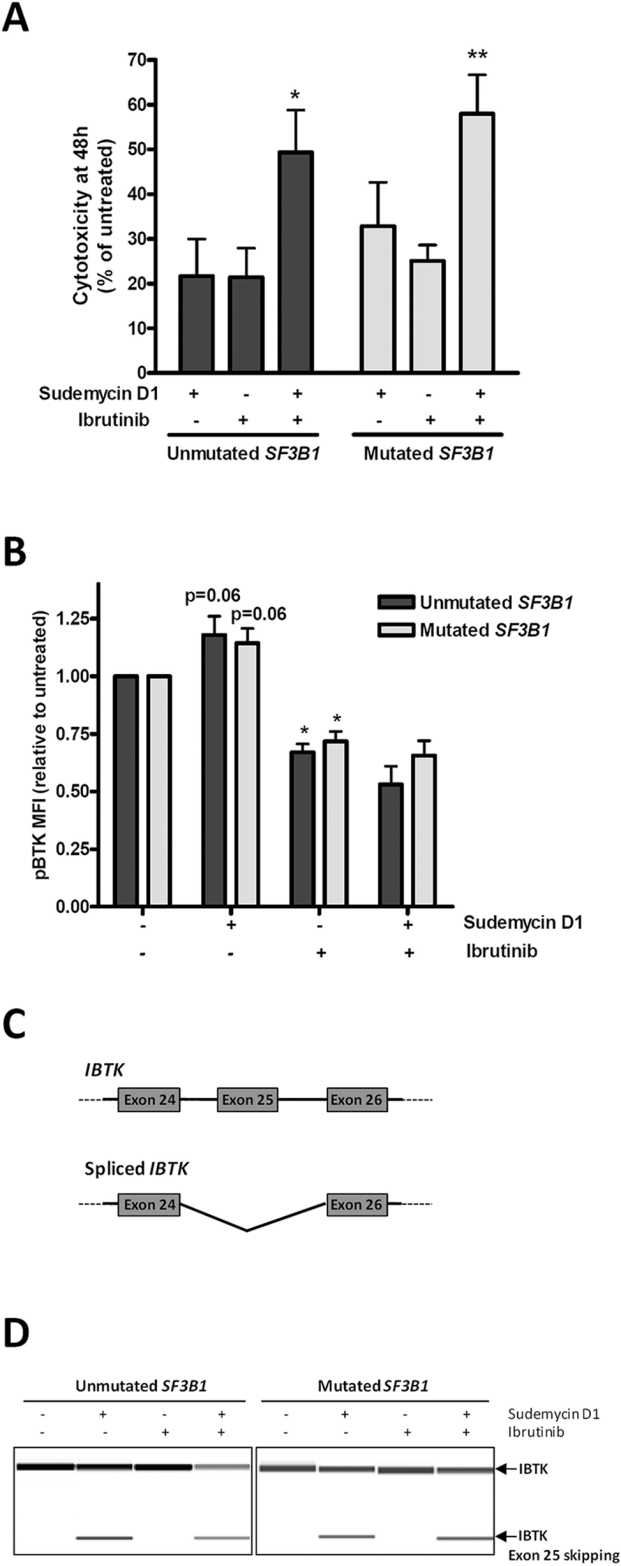
Sudemycin and ibrutinib combination in CLL cells **A.** Cytotoxicity was analyzed after simultaneous sudemycin D1 (100 nM) and ibrutinib (1 μM) exposure for 48 h by annexin V/PI staining. Mean ± SEM of *SF3B1*-unmutated (*n* = 8) and -mutated (*n* = 10) cases. *p < 0.05; **p < 0.01. **B.** Flow cytometry staining of pBtk after exposing *SF3B1*-unmutated (*n* = 5) and -mutated (*n* = 5) CLL cells to sudemycin D1 (250 nM) and ibrutinib (1 μM) for 6 hours. Bars represent mean ± SEM. *p < 0.05. **C.** Diagram representing spliced and unspliced *IBTK* isoforms. Primers were designed against exon 24 and 26 from *IBTK*. **D.** Representative cases of RT-PCR analysis of spliced and unspliced *IBTK* after CLL cells incubation with sudemycin D1 (250 nM) for 6 hours. PCR products were analyzed in a QIAxcel capillary electrophoresis device.

In CLL, the promising results at clinical level of the Btk inhibitor ibrutinib have motivated numerous trials in combination with chemotherapy or immunotherapy [27]. In order to find out a potential clinically relevant combination for sudemycin, we explored the combination with ibrutinib. For this purpose, primary CLL cells from *SF3B1*-unmutated and -mutated cases were simultaneously exposed to sudemycin D1 and the Btk inhibitor for 48 hours. Interestingly, the combination induced significantly more apoptosis than each drug alone both in mutated and unmutated CLL cases (Figure 5A; *p < 0.05, **p < 0.01). Next, we postulated that Btk activity might be synergistically downmodulated with the drug combination. To test this, we analyzed activated phospho-Btk (pBtk) by flow cytometry. Surprisingly, sudemycin D1 induced a moderate increase of pBtk levels both in *SF3B1*-mutated and unmutated CLL cases (*p* = 0.06, marginally significant). As expected, the Btk inhibitor ibrutinib downregulated pBtk (*p < 0.05) while the levels of pBtk in the combination remained as low as ibrutinib alone (Figure 5B), indicating that ibrutinib blocked this undesired effect of sudemycin.

Ibtk is a physiological inhibitor of Btk and particularly its exon 25 encodes the region of the protein required for Btk binding [28, 29]. For this reason, we analyzed whether this exon could be lost by alternative splicing after sudemycin D1 exposure. Consistently, using an RT-PCR strategy to detect *IBTK* transcripts spanning exons 24 to 26, a band of lower molecular weight than the full isoform was amplified after incubation of CLL cells with sudemycin D1 for 6 hours. This shorter band corresponded to the isoform lacking exon 25 as confirmed by sequencing (Figure 5C and Supplementary Information). In contrast, ibrutinib alone did not induce this isoform, which was nevertheless generated with the drug combination (Figure 5D).

Altogether these results demonstrated that the combination of sudemycin D1 with ibrutinib resulted in an enhanced cytototoxic effect involving *IBTK* splicing modulation.

## DISCUSSION

Mutations or misregulated expression of the components of the spliceosome can influence the splicing pattern of several genes and play a crucial role in the development of tumors. In this line, genes encoding the spliceosome have been reported to be frequently mutated or overexpressed in cancer [15, 30, 31]. In CLL, *SF3B1* mutations have emerged as one of the most frequent driver aberrations [6, 8, 32]. Although the functional impact of this alteration in CLL pathogenesis remains still ill-defined, its relevance is highlighted by the evidence that *SF3B1*-mutated patients show more aggressive disease and shorter survival than unmutated ones. In fact, the presence of *SF3B1* mutations is associated with disease subtype [6, 8], progression [6, 9, 33], chemotherapy resistance [9, 14] and overall patient survival [34]. Accordingly, the frequency of *SF3B1* mutations at diagnosis is around 5%–7%, and raises up at progression (17%) or after treatment (12%–24%) [35]. Recently, gene targeting and knockdown experiments collectively have revealed that *SF3B1* is a proto-oncogene [36, 37], and others have proposed that *SF3B1* mutations in CLL lead to a defective DNA-damage response [38]. It is plausible that *SF3B1* mutations alter features of branch point recognition by the SF3B complex. In fact, increased intron retention, alternative splicing and activation of cryptic 3′ splice sites were reported in CLL samples harboring *SF3B1* mutations [6, 8, 39]. Additionally, recurrent mutations in other genes of the mRNA splicing, processing and transport machinery are also found in CLL [10]. Therefore, the evident relevance of the alternative splicing pathway in CLL offers the opportunity to design new specific therapeutic strategies targeting the spliceosome.

In this article, we tested the antitumor activity of the novel splicing modulators sudemycins in CLL primary samples. Sudemycins effectively disturb alternative splicing in CLL cells, similar to what has been observed in other tumor models [20–22]. Our results elucidate for the first time that low doses of these compounds induce apoptosis in CLL cells, while affecting cell death and survival pathways. Intriguingly, CLL cells are exceptionally sensitive to the drug when compared with tumor cells from other B-lymphoid malignancies, with no alterations in *SF3B1* reported (MCL, FL and MM), suggesting that this pathway is crucial for CLL pathogenesis. In this line, and in contrast to normal B cells, CLL cells have increased SF3B1 expression [9]. Accordingly, we demonstrated that the cytotoxic effect is selective for B-CLL cells, given that normal T and B counterparts remain almost unaffected. This tumor-selective toxicity is consistent with a model where the tumor cells have a deficient regulation of alternative splicing, in contrast to normal cells, where this system is highly regulated and in consequence, less sensitive to spliceosome modulators [40]. Several natural products, their derivatives and synthetic analogs that target the spliceosome have showed antitumor properties. This effect has been related to the ability of these drugs to alter the alternative splicing of genes that are relevant for cancer progression [30]. In this context, we showed that the apoptotic effect of sudemycin might be related to *MCL1* alternative splicing, similar to what has been described for other spliceosome modulators [41, 42]. In CLL, Mcl-1 is a key controller of cell survival and correlates with other poor prognostic markers [43]. Sudemycin switches the alternative splicing of *MCL1* toward its proapoptotic short isoform, which presumably leads to the induction of the mitochondrial apoptotic pathway and the activation of caspase-dependent factors. Recently, *MCL1*-_LONG_ down-regulation rather than *MCL1*-_SHORT_ up-regulation has been postulated to be the driver of preferential killing of Mcl-1-dependent cells [44]. These authors demonstrated that cytotoxicity can be rescued by overexpression *MCL1*-_LONG_, whereas *MCL1*-_SHORT_ overexpression had no significant effect on cells. On the other hand, splicing modulators that induced very high levels of *MCL1*-_SHORT_ mRNA in the absence potent *MCL1*-_LONG_ down-regulation exhibited minimal cytotoxicity [44].

Remarkably, we also demonstrated sudemycin efficacy in the *in vivo* setting, specifically in a NSG mice engrafted with peripheral blood cells from CLL patients that recapitulates the role of the human lymph node for CLL cells [45]. Sudemycin reduces the presence of tumor CLL cells in the peripheral blood and spleen of mice both in *SF3B1*-unmutated and -mutated cases though using a very low dose, suggesting that sudemycin might target CLL cells in their protective niches, where several prosurvival pathways are more activated [24]. Among them, NF-κB signaling plays a critical role in CLL pathogenesis and has become a potential therapeutic target [25]. In this context, sudemycin also functionally disturbs NF-κB pathway together with the induction of a spliced *RELA* variant that has a diminished DNA-binding ability [26]. As a consequence, sudemycin treatment inhibits RELA activity, both basal and induced by CD40L-IL4 stimulus, and decreases the expression of several target genes related to inflammation and tumor invasion, such as *IL8*, *MMP9* and *CCL4*. All these results suggest that sudemycin could disrupt tumor microenvironment interactions that are often related to chemoresistance and disease relapses [46].

Importantly, we also showed for the first time that the presence of mutations in *SF3B1* or other spliceosome/RNA processing-related genes confers further sensitivity to sudemycin. According to our results, NF-κB pathway inhibition might at least partially contribute to these differences in sensitivity. While all CLL samples are particularly sensitive to the compound, very low doses would be enough in mutated cases, probably reflecting an accentuated deregulation of the splicing/RNA-processing mechanisms in the mutated cells. In this sense, RNA sequencing analysis of *SF3B1*-mutated CLL cases has revealed a major disturbance in the splicing fidelity of a large number of genes in comparison to -unmutated cases [39, 47].

In CLL, activity and tolerability at clinical level of the Btk inhibitor ibrutinib has led to its approval and has motivated numerous trials in combination with chemotherapy or immunotherapy [27]. In this line, spliceosome modulators in combination with this agent might be an attractive therapeutic strategy to test in CLL patients. Our findings demonstrated for the first time a collaborative antitumor effect of sudemycin with the Btk inhibitor. Although other splicing events in the components of the B-cell receptor pathway might also occur, we have demonstrated that Ibtk might play a crucial role in the interaction between drugs. Ibtk is an important negative regulator of Btk, which leads to inefficient autophosphorylation of Btk on residue Y223 when bound to the PH domain of Btk, thus inhibiting Btk activity [28]. *IBTK* alternative splicing modulation by sudemycin triggers the loss of regulatory function of this protein over Btk, consequently upregulating pBtk that is easily overcome by ibrutinib addition. Our results suggest that increased levels of pBtk after sudemycin exposure could confer enhanced sensitivity to ibrutinib. This would be consistent with a report where high pBtk levels correlated with major sensitivity toward ibrutinib in acute myeloid leukemia [48].

In summary, we show that sudemycin interferes in the splicing program of several genes critical for CLL survival and apoptosis. Our results confirm that targeting the spliceosome in CLL is a promising therapeutic strategy, not only for those patients harboring mutations in the splicing machinery. Collectively, our study provides solid rationale for future clinical development of splicesome inhibitors alone or in combination with ibrutinib in CLL patients.

## MATERIALS AND METHODS

### Isolation and culture of primary cells

Primary cells from 41 CLL patients, who were either untreated or had not received treatment for the at least previous 12 months, were studied (Table 1). Mutations in *SF3B1* and/or other genes involved in mRNA splicing, processing and transport (*U2AF2*, *SFRS2*, *SRSF7*, *XPO1*, *NXF1*, *DDX3X*, *CPEB3*, *SMG7*, *CDCL*, *HELZ*) were known from sequencing studies previously reported [6]. The *IGVH* gene mutational status was determined according to European Research Initiative on CLL (ERIC) guidelines [49]. Percentage of tumor cells (CD19^+^, CD5^+^) together with cytogenetic alterations were obtained from the clinical healthcare activity and assessed by flow cytometry and (FISH) respectively, when sampling. Samples from 8 MCL, 4 FL and 4 MM patients (Supplementary Table S1) and 5 healthy donors were also studied.

Primary cells were isolated from PB (for CLL, MCL and healthy donors) or bone marrow (BM; for MM) by Ficoll-Paque sedimentation (GE Healthcare). Primary FL cells were obtained from lymph nodes (LN) after squirting with RPMI 1640 (Life Technologies) culture medium using a fine needle. Samples were stored within the Hematopathology collection of our institution registered at the Biobank from Hospital Clínic-IDIBAPS (R121004-094). The ethical approval for this project including the informed consent of the patients was granted following the guidelines of the Hospital Clínic Ethics Committee (IRB).

Cells were cultured in RPMI 1640 supplemented with 10% fetal bovine serum (FBS), 2 mM glutamine and 50 μg/ml penicillin-streptomycin (Life Technologies), in a humidified atmosphere at 37°C containing 5% carbon dioxide.

### Drug incubation and analysis of apoptosis

Cells were incubated with 100 to 500 nM of sudemycin C, D1 or D6 (synthesized and formulated in the laboratory of Thomas R. Webb) [22] and/or 1 μM of Ibrutinib (Selleck Chemicals) for 30 minutes to 48 hours. When specified, cells were preincubated for 1 hour with 10 μM of the pan-caspase inhibitor Q-VD-OPh (Calbiochem), or CD40L (1 μg/mL) and IL4 (20 ng/mL; Sigma-Aldrich). Apoptosis was quantified by labeling PS residues exposure with annexin V-fluorescein isothiocyanate (FITC) and nuclei with propidium iodide (PI; eBioscience). For the analysis of apoptosis in CD3^+^ (T lymphocytes) and CD19^+^ (B lymphocytes) populations, cells were simultaneously labeled with anti-CD3-FITC, anti-CD19-Phycoerythrin (PE; Becton Dickinson) and annexin V-Pacific Blue (PB; Life Technologies). All samples contained > 80% of tumor cells except for MM, where tumor cells were co-labeled with anti-CD38-PE (Becton Dickinson) and annexin V-FITC, in order to gate the population of interest. Caspase-3 activation was quantified with CellEvent caspase 3/7 green detection reagent (Life Technologies). Ten thousand single cell events per sample were acquired in an Attune acoustic cytometer (Life Technologies). Cytotoxicity values were obtained after relative quantification with each respective control sample (without treatment). Untreated control sample represented 0% of cytotoxicity for each patient's sample.

### RNA splicing analysis by RT-PCR and sanger sequencing of splicing isoforms

Total RNA was isolated from 5–10 × 10^6^ CLL cells using TRIzol reagent (Life Technologies) according to manufacturer's instructions. cDNA was obtained from 0.5–1 μg of DNA-free RNA with the M-MLV reverse transcriptase (Life Technologies). Subsequently, PCR was run from 2 μl of cDNA with the following reaction setup: 1.25 U Amplitaq Gold DNA Polymerase, 2 mM MgCl2 (Life Technologies), 0.2 mM dNTPs (GE Healthcare) and 0.2 μM of each primer; with temperature cycling conditions of 94°C for 5 minutes, 35 cycles of 94°C for 30 seconds, 60°C for 30 seconds and 68°C for 2 minutes, and finally 72°C for 5 minutes. PCR were performed with the following primer pairs: 5′-GAACCAAAATCACTTTCCCCAAGGAAGG-3′ and 5′-AATGAGGTCCCCACGTTTCTCGGGTGT-3′ for *DNAJB1* [16], 5′-GAGGAGGAGGAGGACGAGTT-3′ and 5′-ACCAGCTCCTACTCCAGCAA-3′ for *MCL1*, 5′-GATGAGATCTTCCTACTGTGTGACAAG-3′ and 5′-GGGAAGGCACAGCAATGC-3′ for *RELA*, 5′-TGTGGATCTCAGAACTATCATGGAA-3′ and 5′-GCCTGAACTATGGCTAGTAACAGACTT-3′ for *IBTK*. PCR products were separated in a QIAxcel capillary electrophoresis device (QIAgen). For sequencing, splicing PCR was performed with the above primers harboring an M13 5′-tail and the following conditions: 95°C for 10 minutes, 10 cycles of 95°C for 30 seconds, 65°C (decreasing 1°C each cycle) for 30 seconds and 72°C for 1 minute, 25 cycles of 95°C for 30 seconds, 55°C for 30 seconds and 72°C for 1 minute, and finally 72°C for 7 minutes. PCR products were separated in an agarose gel and the candidate product was purified with NucleoSpin Gel and PCR Clean-up (Macherey-Nagel). Sequencing PCR was performed with the M13 universal primers and the BigDye Terminator v1.1 Cycle Sequencing Kit (Life Technologies). The experiment was repeated 3 times and representative cases are shown.

### Protein analysis

Protein extracts were obtained and processed as previously described [50]. Membranes were blocked with 2.5% phosphoBlocker (Cell Biolabs) in Tris-Buffered Saline (TBS)-Tween 20. For protein immunodetection, the specific primary antibodies were used: Mcl-1, phospho(Ser536)-p65 (p-p65; Cell Signaling Technology) and β-actin (Sigma-Aldrich). Anti-rabbit and anti-mouse horseradish peroxidase-labeled IgG (Sigma-Aldrich) were used as secondary antibodies. Chemiluminescence was detected with ECL substrate (Pierce Biotechnology) on a mini-LAS4000 Fujifilm device (GE Healthcare). Signal was quantified with Image Gauge densitometric software (Fujifilm) and referred to the respective untreated control.

### Nuclear protein extracts and NF-κB p65 DNA-binding analysis

Cells were incubated with 10 mM pH 7.9, 10 mM KCl, 1.5 mM MgCl_2_, 1 mM DTT supplemented with protease inhibitors (0.5 mM phenylmethylsulfonyl fluoride, 5 μg/ml aprotinin and 2 μg/ml leupeptin; Sigma-Aldrich). Lysis was proceeded by adding 0.6% of NP-40. Nuclei were pelleted and lysed in 20 mM HEPES pH 7.9, 0.46 M NaCl, 1 mM EDTA, 1 mM EGTA, 1 mM DTT supplemented as above. Finally, samples were sonicated at high power in a bath sonicator (Diagenode). NF-κB p65 activity of 2 μg of nuclear protein was determined with the TransAM® NF-κB p65 kit (Active Motif), a DNA-binding ELISA coated with oligonucleotides containing the NF-κB consensus sequence, according to the manufacturer's protocol. Chemiluminescence was read on a mini-LAS4000 Fujifilm device. Signal was quantified with Image Gauge densitometric software (Fujifilm) and referred to the respective untreated control.

### Quantitative real-time PCR

Total RNA was isolated from CLL cells and retrotranscribed as above. The following TaqMan Gene Expression Assays (Life Technologies) were used: *IL8*, *MMP9*, *CCL4* and *ACTB*. The relative expression was quantified by the comparative cycle threshold (*C*_t_) method (ΔΔ*C*_t_), using *ACTB* as endogenous control. Expression levels are given in arbitrary units, referring to the calibrator untreated cells sample.

### Phospho-Btk staining

CLL cells were stained with the Live/Dead® Fixable Aqua Dead Cell Stain Kit (Life Technologies) and blocked with 10% mouse serum (Sigma). Then, samples were CD19-FITC labeled and fixed with 1% paraformaldehyde (Aname). Cell membrane permeabilization was achieved by adding 70% ethanol (Panreac) for 2 hours at −20°C. Intracellular unspecific staining was blocked with 10% mouse serum. Finally, cells were stained for phospho(Y223)-Btk-PE (pBtk) or mouse IgG1 isotype-PE (Becton Dickinson) and 10000 viable CLL cells were analyzed in an Attune acoustic cytometer (Life Technologies). Median Fluorescense Intensity (MFI) of the isotype was subtracted to respective pBtk stained sample and referred to the corresponding untreated sample.

### NSG/CLL mouse xenograft

Sudemycin D6 was tested for *in vivo* efficacy in an adoptive transfer mouse model of CLL, as described by Herman *et al* [45] with modifications. Primary cells from CLL patients (50 × 10^6^ per mice; percentage of tumor cells: 93–99%) were intravenously inoculated through tail vein in six- to eight-week old NSG mice (Charles River Laboratories International, Inc), according to a protocol approved by the animal testing ethical committee of the University of Barcelona (Barcelona, Spain). Six CLL cases (*n* = 3 *SF3B1*-mutated, *n* = 3 *SF3B1*-unmutated) were injected in the mice. Each CLL case was injected in 4 mice (*n* = 2 control; *n* = 2 sudemycin). Mice were treated intravenously with sudemycin D6 (14 mg/kg) or vehicle (10% (2-hydroxypropyl)-β-cyclodextrin in PBS) during 4 days and sacrificed after 5 days of inoculation. Then, spleen and PB samples were recovered and processed as previously described [45]. Cell suspensions were washed, resuspended with 0.5% BSA in PBS and blocked with 10% mouse serum. Samples were incubated with the following anti-human antibodies: CD45-Pacific Blue (PB; Life Technologies), CD19-PE and CD5-FITC (Becton Dickinson) together with PI to assess cell viability. The abundance of CLL cells was counted on an Attune cytometer as the number of viable CLL cells (CD45^+^, CD19^+^ and CD5^+^) per organ (spleen) or per μL (PB).

### Statistical analyses

Data are represented as mean ± SEM of all cases. Statistics were calculated using GraphPad Prism 4.0 software (GraphPad Software). Nonparametric Wilcoxon signed rank test was used to compare the mean of a set of samples to a theoretical value. Comparison between two groups of samples was evaluated by non-parametric Wilcoxon paired or Mann-Whitney *t*-test. Results were considered statistically significant when *p*-value < 0.05 (*p < 0.05, **p < 0.01, ***p < 0.001).

## SUPPLEMENTARY DATA AND TABLE


