# Supplementary material for: The splicing modulator sudemycin induces a specific antitumor response and cooperates with ibrutinib in chronic lymphocytic leukemia
Source: Oncotarget. 2015 Jun 8;6(26):22734–49. doi: 10.18632/oncotarget.4212 (PMC4673195; doi:10.18632/oncotarget.4212)
Supplement: Supplementary file 1 [file oncotarget-06-22734-s001.pdf]

SUPPLEMENTARY DATA AND TABLE

Results

RELA sequence

Consensus sequence

GATGAGATCTTCCTACTGTGTGACAAGGTGCAGAAAGGTATACATCAGGAGGCAGGGGTGGGCTCT
TGGGAGCAAGGGGTGAAGCTGAGCAGAGAAGTGGAGTGTCAGGTAACTGGCTTTGGATACTTCCTCCTGTGCC
TCGGGGGCCTCAGGAGGACATTGAGGTGTATTTCACGGGACCAGGCTGGGAGGCCCGAGGCTCCTTTTCGCAA
GCTGATGTGCACCGACAAGTGGCCATTGTGTTCCGGACCCCTCCCTACGCAGACCCCAGCCTGCAGGCTCCTGT
GCGTGTCTCCATGCAGCTGCGGCGGCCTTCCGACCGGGAGCTCAGTGAGCCCATGGAATTCCAGTACCTGCC
AGATACAGACGATCGTCACCGGATTGAGGAGAAACGTAAAAGGACATATGAGACCTTCAAGAGCATCATGAA
GAAGAGTCCTTTCAGCGGACCCACCGACCCCCGGCCTCCACCTCGA CGCATTGCTGTGCCTTCCC

Legend: Exon 7 Exon 8 Exon 9 Exon 10

Sudemycin-induced isoform sequence

GATGAGATCTTCCTACTGTGTGACAAGGTGCAGAAAGACGATCGTCACCGGATTGAGGAGAAACGT
AAAAGGACATATGAGACCTTCAAGAGCATCATGAAGAAGAGTCCTTTCAGCGGACCCACCGACCCCCGGC
CTCCACCT CGACGCATTGCTGTGCCTTCCC

Legend: Exon 7 Exon 9 Exon 10

IBTK sequence

Consensus sequence

TGTGGATCTCAGAACTATCATGGAAATAGAAGAAAGTAGACAAAAATGTGGAGCTACACCAAAGTCAC
ATTTAGGCAAAACAGTTTCTCATGGAGTTAAACTTTCTCAGAAGCAACGAAAAATGATTGCATTGACTA
CCAAGGAAAACAATTCAGGAATGAATAGCATGGAAACAGTTTTATTCACTCCTTCAAAAGCCCCCAAACC
AGTGAATGCATGGGCATCTTCTCTGCATTCAGTTTCATCCAAGTCATTCCGGGATTTCTTACTAGA AGA
AAAAA AGTC

Legend: Exon 24 Exon 25 Exon 26

Sudemycin-induced isoform sequence

TGTGGATCTCAGAACTATCATGGAAATAGAAGAAAGTAGACAAAAATGTGGAGCTACACCAAAGTC
ACATTTAGGGGCATCTTCTCTGCATTCAGTTTCATCCAAGTCATTCCGGGATTCTTACTAGAAGAAAAAAAGTC

Legend: Exon 24 Exon 26

Supplementary Table S1: Characteristics of other hematological neoplasms samples

Patient *n*º.	Disease	Source	% tumoral cells†	% Cytotoxicity Sudemycin D1 250 nM (24 h)
**MCL1**	MCL	PB	88	10.8
**MCL2**	MCL	PB	94	4.1
**MCL3**	MCL	PB	93	74.9
**MCL4**	MCL	PB	80	15.0
**MCL5**	MCL	PB	96	46.9
**MCL6**	MCL	PB	97	27.1
**MCL7**	MCL	PB	85	21.0
**MCL8**	MCL	PB	92	1.2
**FL1**	FL	LN	81	2.95
**FL2**	FL	LN	84	11.3
**FL3**	FL	LN	80	23.2
**FL4**	FL	LN	80	34.8
**MM1**	MM	BM	50	4.7
**MM2**	MM	BM	74	0.0
**MM3**	MM	BM	60	0.0
**MM4**	MM	BM	45	8.0

MCL, Mantle cell lymphoma; FL, Follicular lymphoma, MM, multiple myeloma
PB, peripheral blood; LN, lymph node; BM, bone marrow
†quantified by flow cytometry
